# Vagus nerve stimulation combined with nerve rehabilitation therapy for upper limb paralysis after hemorrhagic stroke: a stroke-related epilepsy case

**DOI:** 10.1186/s42494-024-00198-9

**Published:** 2025-02-15

**Authors:** Chunsheng Xia, Peng Xu, Lanlan Wang, Dong Zhang, Yinbao Qi, Ming Wu, Ruobing Qian

**Affiliations:** 1 Department of Neurosurgery, The First Affiliated Hospital of USTC, Division of Life Sciences and Medicine, University of Science and Technology of China, 17 Lujiang Road, Hefei, Anhui 230001 PR China; 2 Department of Rehabilitation Medicine, The First Affiliated Hospital of USTC, Hefei, 230001 PR China

**Keywords:** Stroke, Paralysis, SRE, VNS, Rehabilitation

## Abstract

**Background:**

Hemorrhagic stroke has a high incidence, often leaving patients with significant complications such as limb mobility disorders after treatment. Traditional treatment methods for stroke patients mainly include limb function exercises and hyperbaric oxygen therapy, which have shown effective results. In recent years, there have been reports utilizing vagus nerve stimulation (VNS) to treat limb paralysis in ischemic stroke patients, achieving encouraging outcomes. However, there are rare related reports on hemorrhagic stroke.

**Case presentation:**

This report presents a case of a patient who developed left upper limb hemiplegia and recurrent seizures after a hemorrhagic stroke. The patient showed a poor response to standard anti-epileptic treatment and was diagnosed with stroke-related epilepsy. To manage the recurrent seizures, VNS was performed. After the device was activated, the patient reported a significant reduction in abnormal muscle tone and increased mobility impairment in the affected upper limb. Parameters were adjusted, and intermittent stroke electrical stimulation was combined with upper limb rehabilitation exercises. After three months of active treatment, the patient’s seizures were well controlled, and there was significant improvement in upper limb function.

**Conclusions:**

VNS has potential in the rehabilitative treatment of stroke patients with upper limb dysfunction. It is hoped that more patients will benefit from this advanced treatment method in the future, regaining their health and vitality. Additionally, future research needs to further explore the mechanisms and methods of brain remodeling to provide theoretical support and more effective treatment options for stroke patient rehabilitation.

## Background

Stroke, also known as cerebrovascular accident, is a group of acute cerebrovascular diseases characterized by a sudden onset and acute cerebral circulation disorders leading to localized or diffuse brain function deficits. It is classified into ischemic stroke (cerebral infarction) and hemorrhagic stroke (intracerebral hemorrhage). Stroke is associated with high incidence rates, high disability rates, and high mortality rates, causing significant economic loss and severe medical burdens on society. Hemorrhagic stroke has a more acute onset, more severe symptoms, and worse prognosis, with mortality and disability rates far exceeding those of ischemic stroke. Patients often suffer from severe limb paralysis, speech disorders, and other complications, greatly affecting their quality of life.

Stroke-related epilepsy (SRE) refers to seizures that occur within a certain period following a stroke in patients who had no prior history of epilepsy, and after excluding intracranial and systemic diseases [1]. Electroencephalography (EEG) monitoring reveals epileptiform discharges that correspond to the location of the stroke lesion. SRE is a common complication following cerebrovascular events and is a frequent cause of epilepsy in the elderly. The reversible or irreversible brain damage caused by a stroke leading to SRE can prolong the hospital stay for patients with cerebrovascular disease, increase mortality rates, and adversely affect long-term prognosis and quality of life.

Although SRE typically responds to antiseizure medication treatment, 12.9% to 30% of patients may develop drug resistance [2]. For patients with refractory seizures, surgical intervention is the preferred treatment, which may include resection of the epileptogenic focus related to the stroke and neuromodulation techniques [3]. However, in patients with stroke located in functional areas, resecting the epileptogenic focus may further exacerbate motor dysfunction. Therefore, neuromodulation has become the preferred treatment method for drug-resistant SRE.

Recent studies have found that vagus nerve stimulation (VNS), previously used to treat refractory epilepsy, can promote functional remodeling of the cerebral cortex in animals and improve recovery of motor function in ischemic stroke models [4]. After brain injury, cortical functional remodeling occurs, achieving structural and functional reorganization through a series of complex physiological and biochemical processes. Brain remodeling in stroke patients primarily manifests as neuronal regeneration, axonal sprouting, functional reorganization, and synaptic changes, all of which provide possibilities for stroke rehabilitation. Multicenter clinical trials abroad have confirmed that VNS combined with upper limb strengthening rehabilitation significantly improves motor function in patients with upper limb dysfunction after ischemic stroke [5]. In 2021, the Food and Drug Administration (FDA) in the United States approved VNS for treating motor disorders in the limbs following ischemic stroke. However, it has not yet been applied in the field of hemorrhagic stroke treatment. This case report describes a patient with hemorrhagic stroke accompanied by SRE who underwent VNS for the treatment of refractory epilepsy. Postoperatively, there was an improvement in the function of the affected limbs, which is clinically significant, and thus it is reported here.

## Case presentation

**Fig. 1:**
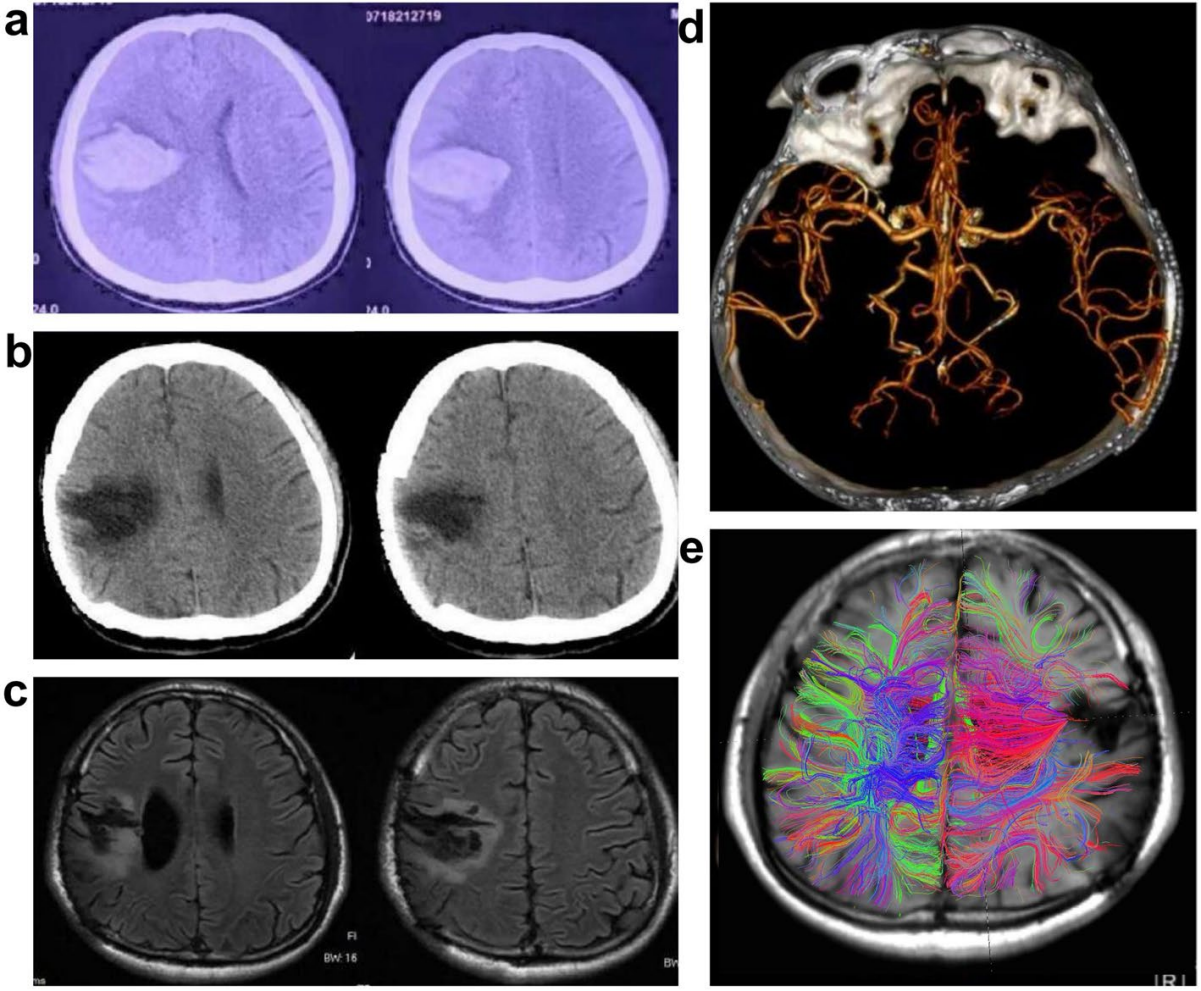
Imaging data of the patient. **a** The initial onset of the disease resulted in bleeding in the right central region. **b** The second surgery involved cranioplasty. **c** The MR T2-Flair suggested postoperative changes in the right central region before the VNS. **d** The cranial computed tomography angiography (CTA) did not reveal any abnormalities such as intracranial aneurysms or vascular malformations. **e** Diffusion tensor imaging (DTI) fiber tract reconstruction indicated interruption of the fiber tract in the right central area

The patient is a 31-year-old male who presented with sudden onset of headache and left-sided hemiparesis. A cranial CT scan indicated hemorrhage in the central region of right frontal lobe, and he was treated conservatively. Subsequently, his symptoms worsened, and the amount of bleeding increased, leading to an emergency right frontal-temporal craniotomy for hematoma evacuation and decompression. Postoperatively, he did not experience limb convulsions, but left-sided hemiparesis persisted. Two weeks after surgery, his left lower limb weakness improved, allowing him to walk, but recovery of the left upper limb was poor, especially with limited flexion and extension of the left hand accompanied by increased muscle tone. He underwent continuous neurological rehabilitation exercises post-surgery. Three months after the cerebral hemorrhage, there was still no improvement in left upper limb weakness or the increased muscle tone, prompting a cranioplasty. Fifteen days post-cranioplasty, the patient experienced left-sided limb rigidity and clonic movements, accompanied by altered consciousness, lasting about 2–3 min, with consciousness recovering approximately 10 min later. This was diagnosed as “SRE” and the patient was prescribed oral sodium valproate (0.2 g, tid). However, he continued to experience seizures, leading to the addition of carbamazepine (0.1 g, tid), oxcarbazepine (0.3 g, bid), and lacosamide (50 mg, bid). Despite these medical treatments, he still experienced intermittent seizures, averaging 2–3 times per month. The seizure manifestations evolved to include clear consciousness accompanied by left-sided limb rigidity and clonus, lasting about 1–2 min, and preceded by a sensation of numbness in the left limb. After one year of active treatment, seizure control remained poor, and he continued to exhibit increased muscle tone in the left upper limb, with the left fingers unable to flex or extend. There were no significant changes in muscle strength or tone before and after each seizure. In May 2024, he was admitted to epilepsy surgery, with physical examination revealing clear consciousness, appropriate responses, equal and round pupils at 3 mm, proximal muscle strength of grade IV in the left upper limb, muscle strength of grade II in the left hand, increased muscle tone in the left upper limb, and normal muscle strength and tone in the left lower limb and right limbs. After admission, he underwent long-term video EEG monitoring, cranial MRI, and other relevant preoperative evaluations for epilepsy (Fig. 1).

On May 22, 2024, the patient underwent VNS implantation under general anesthesia. A seizure occurred again on June 2, 2024. The VNS was activated on June 3, 2024, with a stimulation current of 0.3 mA, a stimulation duration of 30 s, an interval of 5 min, a frequency of 20 Hz, and a pulse width of 250 µs. On June 4, 2024, one day after activation, the patient reported some relief in increased muscle tone of the left upper limb.

After the VNS was activated, the patient was transferred to the rehabilitation medicine department for limb rehabilitation therapy. The stimulation parameters for epilepsy were intermittently adjusted to those suitable for stroke stimulation: a stimulation current of 0.8 mA, a stimulation duration of 0.5 s, an interval of 10 s, a frequency of 30 Hz, and a pulse width of 100 µs. During rehabilitation, continuous stimulation was applied for one hour daily, followed by a return to the epilepsy mode.

Rehabilitation training occurred six times a week, utilizing the Bobath technique and proprioceptive neuromuscular facilitation (PNF) techniques for upper limb physical therapy (PT), combined with functional training for the hemiplegic hand. The Fugl-Meyer Assessment (upper limb) was used to evaluate treatment effects. A total of five assessments were conducted (Table 1): the first assessment before surgery scored 23 points; the assessment one day after activation scored 32 points; the assessment 10 days after activation scored 36 points; and the assessment 42 days after activation scored 40 points, indicating an improvement of 17 points compared to pre-surgery score. This improvement is similar to the results reported by Cummins et al. in their first case report on VNS in patients with hemorrhagic stroke, which noted a 14-point improvement after 6 weeks of intervention [6].

**Table 1:** The assessment of the patient pre- and post- VNS

	Pre-VNS	Post-VNS			
		1 day	10 days	42 days	90 days
FMA-UE	23	32	36	40	43
SAS	70	64	60	52	46
FTHUE	II	V	V	V	V

*Abbreviations*: *FMA-UE* Fugl-Meyer Assessment Upper Extremity, *SAS* Self-rating anxiety scale, *FTHUE* Functional test for the hemiplegic upper extremity

**Fig. 2:**
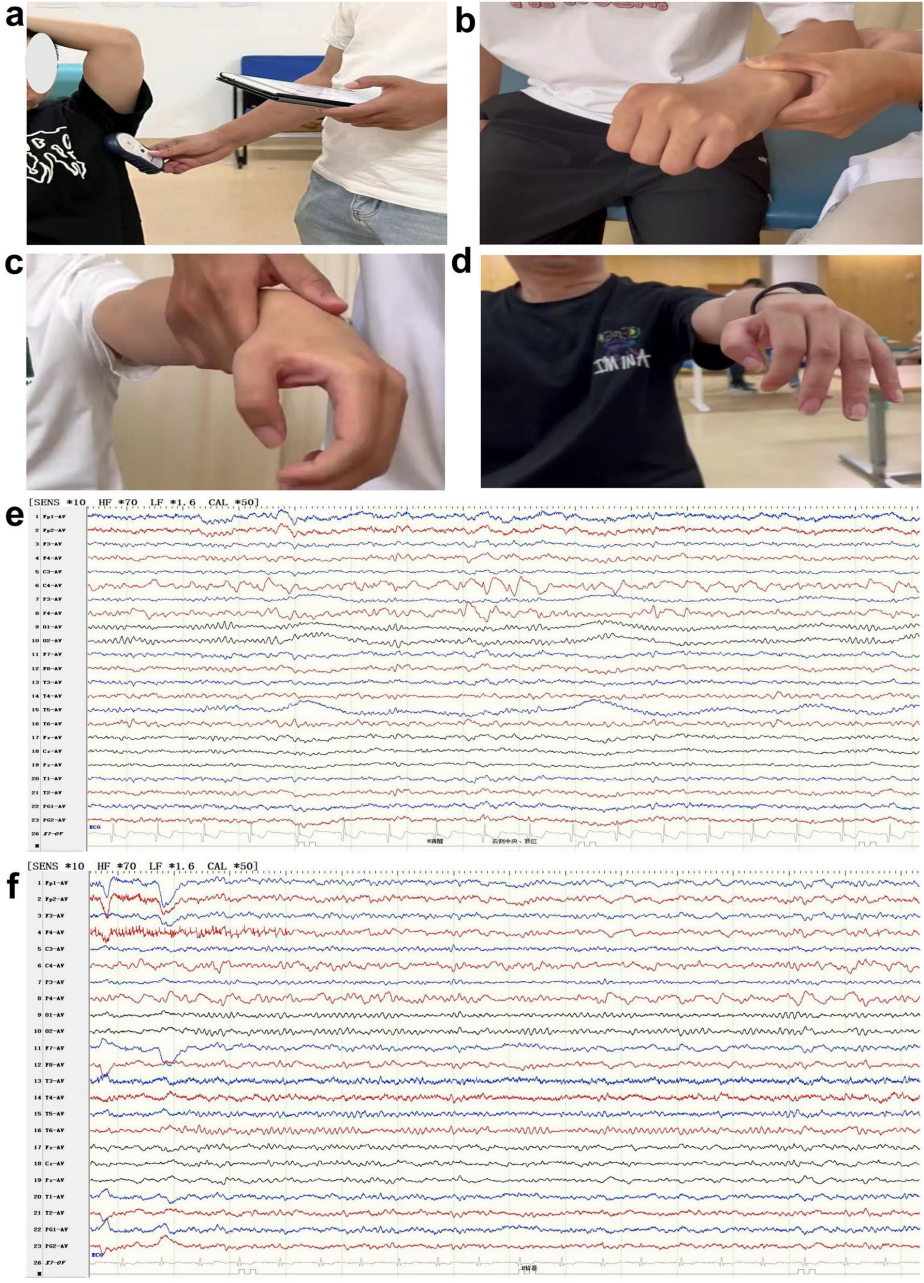
Rehabilitation status of the patient’s right upper limb and improvement in EEG. **a** Before rehabilitation therapy, the doctor adjusted the epilepsy stimulation mode of the vagus nerve stimulator to the stroke mode. **b** Preoperative VNS, the left upper limb required assistance for elevation, and the fingers were contracted and unable to extend. **c** At 42 days post-VNS, the left upper limb still required assistance for elevation, but the muscle tone in the fingers decreased, allowing for partial extension. **d** At 90 days post-VNS, the left upper limb could be elevated independently, and both muscle tone and extension function of the fingers further improved. **e** Preoperative scalp EEG indicated a high amplitude of sharp slow waves in the right central parietal area. **f** At 90 days post-VNS, a follow-up EEG showed a significant reduction in sharp slow waves in the right central parietal area compared to the preoperative measurements

At the 90-day follow-up after stimulation, the patient scored 43 points on the Fugl-Meyer Assessment for the Upper Extremity (FMA-UE), and the Functional Test for the Hemiplegic Upper Extremity (FTHUE) [7] improved from grade II pre-surgery to grade V at present (Fig. 2). Additionally, the self-rating anxiety scale (SAS) assessment score decreased from 70 points pre-surgery to 46 points, indicating the patient is currently in a state of no anxiety. Three months after activation, the patient experienced only one seizure and is currently taking oral sodium valproate extended-release tablets at a dosage of 0.5 g twice a day, with a significant improvement in quality of life. He achieved an McHugh classification of grade I (more than 80% reduction in seizure frequency and complete cessation of seizures).

## Discussion

Stroke-induced brain tissue damage is the main cause of the occurrence of SRE. Hemorrhagic stroke leads to the leakage of blood-derived substances (such as hemosiderin or iron) into the brain parenchyma, which can trigger epilepsy by activating transforming growth factor-beta (TGF-β) receptors on astrocytes [8]. SRE is more common in hemorrhagic strokes than in ischemic strokes, suggesting the significant role of iron deposition in the development of post-stroke epilepsy. The deposition of hemosiderin may result in persistent changes in neuronal excitability, leading to inflammation and glial cell proliferation following cerebral hemorrhage, which is closely related to the occurrence of SRE [9]. Additionally, the occurrence of SRE negatively impacts stroke outcomes. It can lead to decreased intracranial blood flow and increased intracranial pressure, thereby exacerbating secondary neurological damage. Patients with stroke combined with SRE tend to have poorer prognoses, and the risk of recurrent stroke is elevated. Therefore, the treatment of SRE secondary to stroke has become a significant and challenging area of research.

VNS is a novel neuromodulation technique primarily used in the clinical treatment of refractory epilepsy. It utilizes implantation of a small pulse generator that sends electrical impulses through electrodes to the left cervical vagus nerve. By stimulating the vagus nerve, it regulates brain networks to suppress abnormal neuronal discharges, thereby controlling seizures and improving comorbidities associated with epilepsy. Research indicates that VNS has the potential to treat various neurological disorders, such as Alzheimer’s disease, Parkinson’s disease, depression, traumatic brain injury, tinnitus, and sleep disorders [10]. Currently, VNS has been approved by the U.S. FDA for the treatment of depression, epilepsy, and ischemic stroke.

A randomized triple-blind controlled trial by Dawson et al. demonstrated that implanted VNS shows promising prospects for application in upper limb rehabilitation after cerebral infarction [5]. In this study, all patients exhibited a significantly greater mean improvement in the FMA-UE (for the upper limb) on the first day after completing VNS combined rehabilitation therapy compared to the control group. At the 90-day follow-up after treatment, 47% of patients in the VNS group showed a clinically significant improvement in FMA-UE scores, which was significantly higher than the 24% improvement observed in the control group. VNS also demonstrated significant advantages in the long-term efficacy for treating upper limb dysfunction following stroke. Another long-term follow-up study included 15 patients with cerebral infarction from three medical centers in the U.S. and one hospital in the U.K., who underwent implanted VNS treatment combined with six weeks of clinical rehabilitation training [11]. The results showed that the significant effects of VNS combined with rehabilitation training at one year could be sustained into the second and third years, with further improvements in upper limb function observed in those years.

Currently, it is believed that VNS can enhance the effectiveness of behavioral-level rehabilitation training, although it does not directly drive improvements in functional outcomes at the behavioral level. Animal studies have also indicated that the combination of VNS and motor training enhances synaptic plasticity in motor-related brain regions and neural networks [12]. Therefore, in practical clinical applications, scientifically designed rehabilitation training (including home training programs) combined with VNS is indispensable. Although clinical research on the application of VNS has progressed rapidly, studies investigating the underlying mechanisms have lagged behind. Despite the long history of VNS application in epilepsy, treatment response rates remain low and exhibit considerable individual variability, the reasons for which are still unclear.

Research by Bowles et al. indicated that VNS, when synchronized with positive reinforcement, can improve motor learning outcomes through cholinergic mechanisms, thereby enhancing the function of the motor cortex [13]. A synaptic tracing study in rats demonstrated that VNS combined with rehabilitation training could double the number of synaptic connections in the corticospinal tract network that regulates motor function compared to rehabilitation training alone [4]. Ekici et al. found that VNS can inhibit inflammatory responses and protect neurons by activating cholinergic anti-inflammatory pathways [14]. Additionally, some studies have indicated that VNS may reduce levels of pro-inflammatory cytokines (TNF-α, IL-1β), alleviating intracranial inflammatory responses [15]. Yang et al. discovered that VNS can protect the blood-brain barrier and plays a critical role in reducing edema, maintaining homeostasis in the central nervous system, and ensuring normal neuronal function [16]. Functional magnetic resonance imaging (fMRI) studies have shown that VNS modulates blood flow and neural activity across a wide range of brain regions, including the brainstem, thalamus, hippocampus, hypothalamus, and neocortex, in a stimulus intensity-dependent manner [17].

In summary, the efficacy of VNS in the treatment of ischemic stroke is well established. The mechanisms underlying this efficacy may enhance neural plasticity and promote recovery of neurological function and regeneration after neural damage through the regulation of inflammatory responses, neurotransmitter levels, and hemodynamics. This case report demonstrates that VNS also plays a positive role in the rehabilitation of limb function in hemorrhagic stroke, suggesting that its mechanisms may be similar to those in ischemic stroke. However, current literature mainly consists of case reports regarding its use in hemorrhagic stroke, lacking support from large sample data, which represents a direction for our future work. Furthermore, there is a need for deeper exploration of its mechanisms of action in post-stroke functional impairments and continuous improvement of its clinical applications.

## Conclusions

VNS has significant potential in the rehabilitative treatment of upper limb dysfunction in patients with hemorrhagic stroke. It is hoped that more patients with hemorrhagic stroke will benefit from this advanced treatment method in the future. Additionally, future research needs to further explore the mechanisms and methods of brain remodeling to provide theoretical support and more effective treatment strategies for the rehabilitation of stroke patients.

## Data Availability

Not applicable.

## Abbreviations

CT: Computed tomography
EEG: Electroencephalogram
FDA: Food and drug administration
FMA-UE: Fugl-Meyer Assessment Upper Extremity
FTHUE: Functional test for the hemiplegic upper extremity
MRI: Magnetic resonance imaging
PNF: Proprioceptive neuromuscular facilitation
PT: Physical therapy
SAS: Self-rating anxiety scale
SRE: Stroke-related epilepsy
VNS: Vagus nerve stimulation
